# The ANOCA/INOCA Dilemma Considering the 2024 ESC Guidelines on Chronic Coronary Syndromes

**DOI:** 10.3390/jcdd11100302

**Published:** 2024-10-01

**Authors:** Vincenzo Sucato, Cristina Madaudo, Alfredo Ruggero Galassi

**Affiliations:** Division of Cardiology, Department of Health Promotion, Mother and Child Care, Internal Medicine and Medical Specialties (ProMISE), University Hospital Paolo Giaccone, University of Palermo, Via del Vespro n 129, 90127 Palermo, Italy

## 1. Introduction

Cardiovascular disease remains a significant cause of morbidity and mortality worldwide, and its manifestations continue to pose a challenge in clinical practice [1]. Currently, great interest is focused on the phenomenon of angina and ischemia in patients without obstructive coronary disease [2,3]. The very recent ESC 2024 guidelines on chronic coronary syndromes have given significant space to this topic, focusing on diagnosing and managing these diseases [4]. ANOCA is angina with non-obstructive coronary arteries, while INOCA is ischemia with non-obstructive coronary arteries [4]. Despite advances in diagnostic techniques and treatments for coronary artery disease (CAD), a significant subset of patients, particularly women, continue to experience symptoms of angina and ischemia without the typical signs of obstructive lesions in the epicardial coronary arteries [5]. This pathology represents a large problem, as the quality of life (QoL) of patients suffering from recurrent angina is very poor, and hospitalizations are numerous, as are the healthcare costs [6]. Among patients referred for coronary angiography because of symptoms of angina, a significant proportion, up to 70%, do not have obstructive coronary artery disease [7,8]. Of these, approximately 25% have demonstrable ischemia, known as INOCA. Women are disproportionately affected by this condition, with 50%-70% of the women referred for invasive coronary angiography (ICA) presenting with non-obstructive coronary arteries, compared with 30–50% of men [5].

Coronary microvascular dysfunction (CMD) and vasospastic angina (VSA) are key contributors to symptoms in this patient population. While traditional CAD is concentrated in the major epicardial arteries, patients with ANOCA/INOCA often experience dysfunction arising from the smaller coronary vessels or vasospastic activity in the larger arteries [9].

The underlying manifestation of these conditions comprises the following:

- CMD is characterized by the inability of the coronary microvasculature to correctly dilate in response to increased oxygen demand [10]. This dysfunction may result from structural changes, such as increased microvascular resistance, reduced coronary flow reserve (CFR), and functional problems, such as abnormal vasoconstriction of the coronary arterioles. Research indicates that CMD occurs in 39% to 54% of patients with non-obstructive CAD, depending on the diagnostic techniques used, including positron emission tomography (PET), cardiac magnetic resonance imaging (CMR), or invasive coronary function testing [11,12]. The risk factors for CMD include age, smoking, diabetes, hypertension, and dyslipidemia, with a significant link between CMD and inflammatory conditions such as systemic lupus erythematosus (SLE) and rheumatoid arthritis [13,14,15,16].
- VSA occurs when abnormal vasoconstriction of the epicardial coronary arteries leads to temporary blockages and myocardial ischemia. This dynamic obstruction is often missed during coronary angiography unless provocative testing is performed with agents such as acetylcholine (ACh) [17].

CMD and VSA often occur together, making diagnosis and treatment more challenging. The diagnosis is very complex, as these are the only pathologies in which traditional angiography has low sensitivity in identifying microvascular dysfunction [18]. Therefore, the use of advanced methods such as invasive coronary function testing with Ach and adenosine is not only helpful but crucial to distinguish between the different endotypes of ANOCA/INOCA, including endothelial dysfunction, impaired vasodilation, and epicardial or microvascular spasm.

## 2. Diagnostic and Prognostic Considerations

The diagnostic process for patients with ANOCA and INOCA can be frustrating and prolonged. In most cases, obstructive coronary lesions are not detected using coronary angiography. Blood biomarker detection in patients with suspected CMD and MVA can facilitate the diagnosis of these pathological conditions, offering several advantages, such as reduced costs, less risk of physical harm, greater availability/accessibility, and a prognostic value [19]. The TIMI frame count (TFC) and longitudinal strain have proved to be two valid tools for studying those alterations in the coronary tree not immediately visible or documented by coronary angiography [20]. However, the comprehensive and invasive testing of coronary function has become essential to diagnose these conditions accurately [7].

The CorMicA (CORonary MICrovascular Angina]; NCT03193294) trial evaluated the effect of an interventional diagnostic procedure (IDP) linked to stratified medicine on the health status in 151 patients with angina without obstructive disease [21].

An IDP (including the guidewire-based assessment of coronary flow reserve, microcirculatory resistance index, and fractional flow reserve, followed by an acetylcholine vasoreactivity test) with linked medical therapy is routinely feasible and improves angina in patients without obstructive CAD [21].

Intracoronary imaging could reveal a strategic tool for diagnosing and managing these diseases [22]. This test using Ach administration assesses the endothelial function of the epicardial arteries and provides vital information about the coronary microvasculature. At the same time, adenosine or papaverine can be used to assess impaired vasodilation through measurements of the CFR, the microvascular resistance index (IMR), and other relevant parameters [23]. Once ANOCA or INOCA is diagnosed, understanding the prognosis is equally essential. Even in the absence of obstructive CAD, patients with ANOCA/INOCA face a higher risk of adverse cardiovascular events than those with normal coronary arteries [7]. The coexistence of CMD and VSA leads to poorer prognoses, and specific subgroups, particularly those with reduced CFR or impaired endothelial function, are at even greater risk of significant cardiovascular events.

## 3. Treatment Challenges: The Present and the Future

ANOCA/INOCA presents a considerable challenge due to the heterogeneous nature of the patient population and the intricate mechanisms involved. Patients with these diseases would benefit from personalized care and individualized treatment strategies [24]. Pharmacological therapies such as beta-blockers, calcium channel blockers, angiotensin-converting enzyme inhibitors (ACE-Is), and anti-anginal agents such as ranolazine have been investigated for treating patients with coronary microvascular dysfunction (CMD) or vasospastic angina [25]. These drugs enhance the coronary blood flow, lower the microvascular resistance, and relieve angina symptoms. For those with vasospastic angina, calcium antagonists are typically the first-line treatment, often requiring higher doses for more severe cases [26]. In the case of CMD, therapies that enhance endothelial function and coronary flow, including ACE-Is and ranolazine, have shown encouraging results [27].

Numerous ongoing clinical trials are examining new treatments for ANOCA/INOCA. For example, the WARRIOR trial (NCT03417388) is investigating the effects of intensive statin and ACE-I therapy in women with ANOCA to reduce major adverse cardiovascular events. Likewise, the PRIZE trial (NCT04097314) assesses the effectiveness of Zibotentan, an endothelin A receptor antagonist that aims to address the vasoconstrictor response in coronary microvessels.

## 4. What Did We Know? What Do We Know Today?

**Figure 1:**
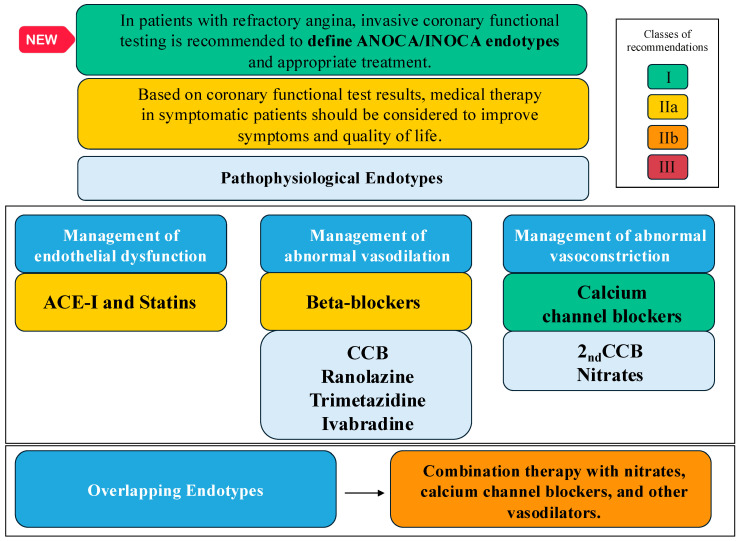
Management of ANOCA/INOCA according to the 2024 ESC Guidelines on chronic coronary syndromes.

The ESC guidelines published in August 2024 on chronic coronary syndromes have re-addressed the ANOCA/INOCA issue [4]. First, assessing the patient’s CAD risk correctly through the Risk-Factor-Weighted Clinical Likelihood Model (I B) is crucial. Furthermore, adjusting the risk estimate through additional clinical data and the CT calcium score is possible. In patients with an uncertain diagnosis according to the noninvasive testing, invasive coronary angiography with the availability of invasive functional assessments is recommended to confirm or exclude the diagnosis of obstructive CAD or ANOCA/INOCA (I B). In terms of treatments, Figure 1 shows current guideline recommendations regarding the management of patients with ANOCA/INOCA. 

## 5. Conclusions

Angina and ischemia in patients with non-obstructive coronary arteries represent a unique and often overlooked challenge in contemporary cardiology. Conditions such as ANOCA and INOCA are prevalent, particularly among women, and carry a significant symptom burden and an increased risk of adverse cardiovascular events. As we deepen our understanding of CMD and VSA mechanisms, the need for in-depth diagnostic protocols and personalized treatment strategies becomes more evident. Adopting a multidisciplinary patient-centered approach may improve the quality of life and long-term outcomes for individuals with these conditions. An endotype-based approach is the answer to managing this challenging patient population.
